# Zinc-Substituted Pheophorbide A Is a Safe and Efficient Antivascular Photodynamic Agent

**DOI:** 10.3390/ph15020235

**Published:** 2022-02-16

**Authors:** Milena J. Szafraniec, Monika Toporkiewicz, Andrzej Gamian

**Affiliations:** 1 Hirszfeld Institute of Immunology and Experimental Therapy, Polish Academy of Sciences, 53-114 Wrocław, Poland; andrzej.gamian@hirszfeld.pl; 2 Łukasiewicz Research Network—PORT Polish Center for Technology Development, 54-066 Wrocław, Poland; monika.toporkiewicz@port.lukasiewicz.gov.pl

**Keywords:** zinc pheophorbide a, chlorophyll derivatives, photodynamic therapy, serum albumin, apoptosis, endothelium, HUVEC

## Abstract

The present study focuses on the photodynamic activity of zinc-substituted pheophorbide a against human endothelial cells. Previously, zinc pheophorbide a has been shown to be a very potent photosensitizer but also a strong albumin binder. Binding to albumin significantly reduces its availability to cancer cells, which may necessitate the use of relatively high doses. Here we show that zinc pheophorbide a is very effective against vascular endothelial cells, even in its albumin-complexed form. Albumin complexation increases the lysosomal accumulation of the drug, thus enhancing its efficiency. Zinc pheophorbide a at nanomolar concentrations induces endothelial cell death via apoptosis, which in many cases is considered a desirable cell death mode because of its anti-inflammatory effect. Additionally, we demonstrate that in comparison to tumor cells, endothelial cells are much more susceptible to photodynamic treatment with the use of the investigated compound. Our findings demonstrate that zinc pheophorbide a is a very promising photosensitizer for use in vascular-targeted photodynamic therapy against solid tumors, acting as a vascular shutdown inducer. It can also possibly find application in the treatment of a range of vascular disorders. Numerous properties of zinc pheophorbide a are comparable or even more favorable than those of the well-known photosensitizer of a similar structure, palladium bacteriopheophorbide (TOOKAD^®^).

## 1. Introduction

Photodynamic therapy is an evolving modality for the treatment of a range of both neoplastic and non-neoplastic diseases. It involves either the systemic or local administration of a photosensitive drug (photosensitizer, PS) followed by site-specific irradiation with visible light of a specific wavelength. This induces either the electron transfer processes (type I reaction) or a formation of highly reactive singlet oxygen (type II reaction), which destroy a diseased tissue [1,2].

A particular variant of photodynamic therapy is vascular-targeted photodynamic therapy (VTP), which uses a PS accumulated not in tumor tissue itself, but in the surrounding blood vessels. Vascular damage due to VTP results in vessel constriction, blood flow stasis, and thrombus formation, which consequently blocks the supply of oxygen and nutrients, causing tumor necrosis [3]. The advantages of VTP over traditional photodynamic therapy include: (1) the use of hydrophilic PSs, which, due to their short retention time, do not generate adverse photosensitization of a patient; (2) the rapid localization of a PS in endothelium; (3) the availability of molecular oxygen required for a photochemical reaction; (4) the amplified effect on tumor cells resulting from the supply of multiple tumor cells through a single vessel [3,4]. Currently, three vascular-targeted photosensitizers, namely palladium bacteriopheophorbide (TOOKAD^®^), its water-soluble derivative padeliporfin (WST11), and verteporfin (Visudyne^®^), are approved for clinical use [5,6]. Nevertheless, new vascular-targeted PSs with increased selectivity and efficacy are still being sought. Their design often includes the usage of different types of targeting moieties, such as peptides, antibodies, or nanocarrier systems, which allow acting on specific molecular pathways [3,7].

**Figure 1:**
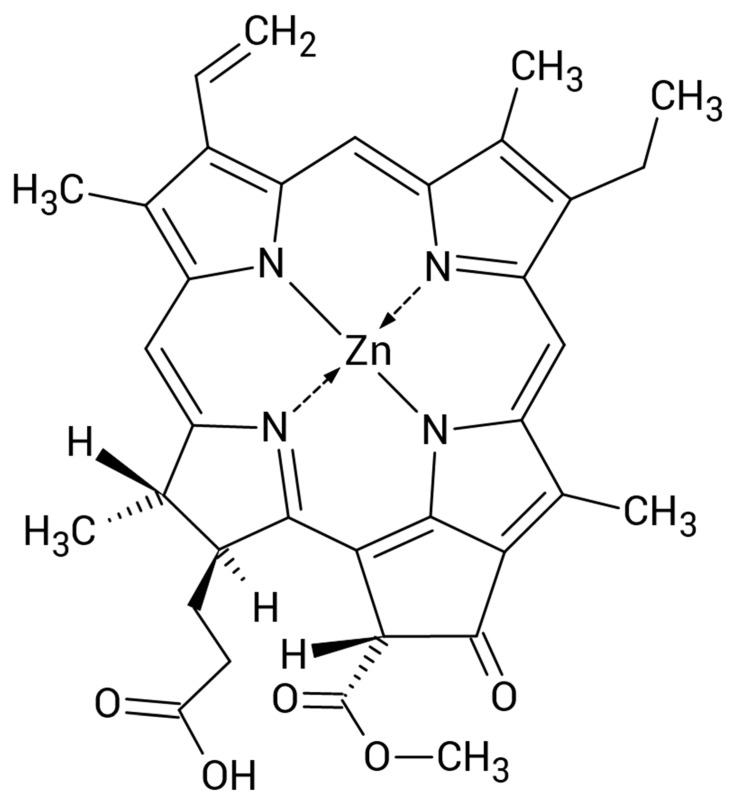
Chemical structure of zinc pheophorbide a.

Pheophorbide a is a natural product of chlorophyll a breakdown, formed via its demetallation and the removal of phytol. Zinc pheophorbide a (Zn-Pheide, Figure 1) is a semi-synthetic derivative of pheophorbide a with a much higher water solubility compared to its precursor [8]. It possesses excellent photodynamic properties, including strong light absorption in the therapeutic window of human tissue, low dark toxicity, and high efficiency of reactive oxygen species generation [8,9,10,11,12]. Zinc pheophorbide a has already been shown to exert a very strong photodynamic effect against human adenocarcinoma and mouse melanoma in vivo [10,11]. On the other hand, however, in our previous studies we showed that Zn-Pheide binds strongly to albumin, which significantly impairs its uptake by tumor cells, thus reducing it efficacy [13,14]. Considering that, we hypothesized that the strong photodynamic effect of Zn-Pheide observed in vivo might be due to its high activity against tumor vessels endothelium, since endothelial cells are known to express albumin-binding receptors potentially involved in the uptake of PS–albumin complexes. The most thoroughly characterized albumin receptor is gp60 (albondin), which initiates the process of the caveolin-mediated endocytosis of albumin, in which caveolin-1 plays the major role [15].

In light of the above, a question arose whether the Zn-Pheide–albumin complex is recognized by gp60 and internalized via caveolin-1-mediated endocytosis, similarly to free albumin and albumin-bound paclitaxel (Abraxane^®^) [15,16,17]. Moreover, given the antioxidant properties of albumin, we also considered whether the possible uptake of the PS in its albumin-complexed form would affect its photodynamic activity [18]. Revealing a photodynamic potential of Zn-Pheide against endothelial cells would make it a candidate for a vascular-targeted PS, applicable not only in the treatment of solid tumors but also several vascular anomalies, such as hemangiomas, venous and arteriovenous malformations, as well as capillary malformations known as port-wine stains [19,20].

The aim of the present study was therefore to investigate the photodynamic activity of Zn-Pheide against human endothelial cells, particularly in the context of its naturally occurring complexation with serum albumin and transport via the breast cancer resistance protein (BCRP, ABCG2), which are interrelated [13]. The experiments were carried out using human umbilical vein endothelial cells (HUVECs), being one of the most commonly used model systems to study vascular biology in vitro and shown to perform the transcytosis of albumin via the gp60 receptor and caveolin-1 protein [21]. We evaluated the impact of binding to albumin on the cellular accumulation, localization, and efflux of the PS, as well as on the photodynamic effect induced with its application. In addition, we compared HUVEC cells with human breast carcinoma cells (MCF-7) in terms of their susceptibility to Zn-Pheide-mediated photodynamic treatment (PDT), particularly in the context of the binding of the PS to albumin. Furthermore, we investigated the type of cell death induced by Zn-Pheide both in the presence and absence of albumin. Additionally, based on literature reports, we compared the photophysical and pharmacokinetic parameters of Zn-Pheide with those of other PSs of similar structure used in VTP, thus assessing the applicability of the former in the VTP of cancer and other diseases.

## 2. Results

The experiments on the MCF-7 and HUVEC cell lines with the use of Zn-Pheide were initiated by determining the dark cytotoxicity of the PS. The results of these assays are presented in Figure S1 (Supplementary Materials). The PS at a concentration of 1 µM with a 3 h incubation was found to be nontoxic to both lines, therefore this concentration or lower concentrations were used in subsequent studies.

**Figure 2:**
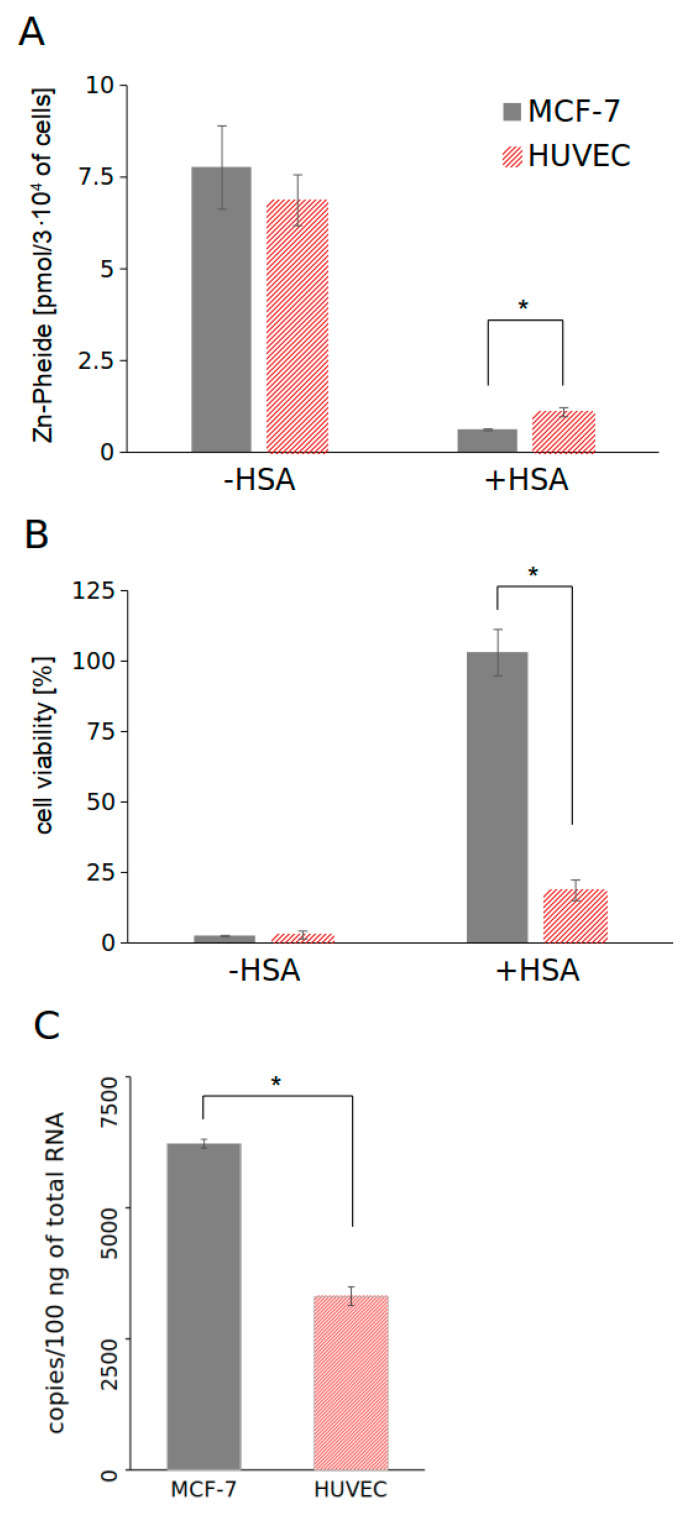
Comparison of cellular accumulation of Zn-Pheide (**A**), cell viability after Zn-Pheide-induced PDT (**B**), and expression of the *ABCG2* gene (**C**) in MCF-7 and HUVEC cells. Cellular accumulation of the PS and cell viability after PDT were measured in the presence and absence of HSA (250 μM). The concentration of Zn-Pheide in both experiments was equal to 1 μM. Expression of BCRP was determined by quantitative real-time PCR. Data are presented as mean ± SD, *n* = 3. Significant differences between HUVECs and MCF-7 cells are indicated by asterisks (*), *p* < 0.05.

In our previous studies, we observed that at a human serum albumin (HSA) concentration 250 times higher than the concentration of Zn-Pheide, saturation occurs, i.e., Zn-Pheide is completely bound to HSA [14]. Therefore, we compared the accumulation and the corresponding PDT effect induced by Zn-Pheide in MCF-7 and HUVEC cells, both in the absence of albumin and at its saturating concentration. The results of these experiments are shown in Figure 2A,B. In the absence of albumin, the accumulation of Zn-Pheide in MCF-7 and HUVEC cells was similar (Figure 2A). At the saturating concentration of HSA, in turn, the level of Zn-Pheide was significantly decreased in both cell lines, although the HUVECs accumulated roughly a two-fold higher amount of the PS than MCF-7. The photodynamic effect induced by 1 µM Zn-Pheide in MCF-7 cells at the applied PDT conditions was completely abolished in the presence of HSA (Figure 2B). On the other hand, in the case of HUVECs, as much as 80% of the cells were killed when Zn-Pheide was complexed with HSA.

The observed decrease in accumulation of Zn-Pheide in the presence of HSA could be due to HSA–PS complex formation as well as the efflux of Zn-Pheide via the BCRP (ABCG2) transporter, which is significantly increased in the presence of albumin [13]. To evaluate the involvement of BCRP in the regulation of the PS level in both cell lines, the expression of this transporter was determined at the mRNA level by quantitative real-time PCR. The expression of BCRP was found to be two-fold lower in HUVECs than in MCF-7 cells (Figure 2C).

**Figure 3:**
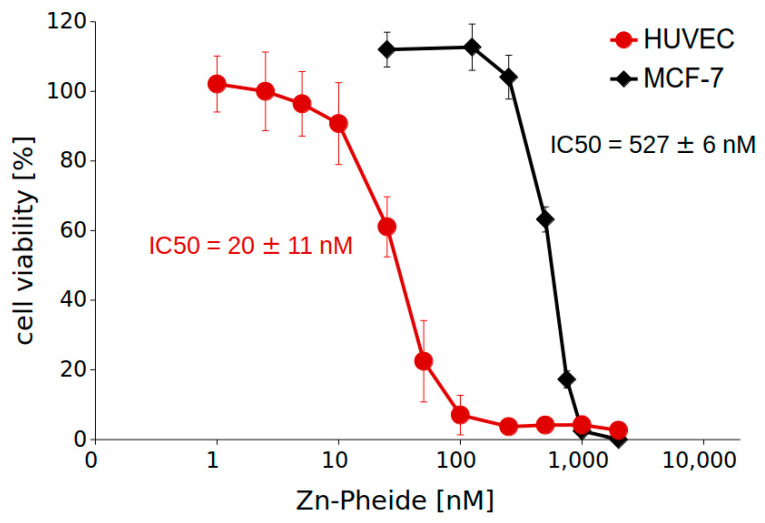
Dose–response curves of MCF-7 and HUVEC cells treated with Zn-Pheide (see the text for details). Cell viability was determined by MTT assay. The results are shown as mean ± SD, *n* = 3.

Lower expression of BCRP in HUVECs compared to MCF-7 cells is undoubtedly one of the factors determining the stronger photodynamic effect observed in these cells in the presence of HSA. However, other HSA-independent factors may also contribute to the higher susceptibility of the former cell line to Zn-Pheide-mediated PDT. To explain this, we examined the photodynamic effect induced with the use of a range of Zn-Pheide concentrations in both cell lines in the absence of HSA and plotted the dose–response curves (Figure 3). Different concentrations of the PS were applied to the cells in order to show the expected sigmoidal shape of the curves.

We found that HUVECs were much more susceptible to Zn-Pheide-induced PDT than MCF-7 cells, regardless of the PS complexation with HSA. The IC50 value was an order of magnitude lower for the former than for the latter cell line. Additionally, a higher slope was observed for MCF-7 than for HUVECs, showing that the cytotoxicity of Zn-Pheide towards the latter cell line increases relatively slowly with increasing concentration of the PS.

**Figure 4:**
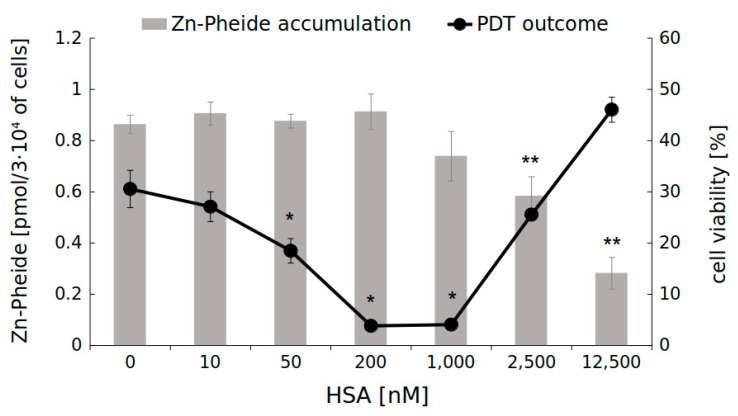
Accumulation of Zn-Pheide (50 nM) in HUVEC cells and the corresponding photodynamic effect at various concentrations of HSA. The results are presented as mean ± SD, *n* = 3. Single asterisks (*) indicate significant differences in cell viability compared to HSA-free cells (*p* < 0.05). Double asterisks (**) indicate significant differences in Zn-Pheide level compared to HSA-free cells (*p* < 0.05).

At high concentrations of HSA (250 µM), required for the complete binding of 1 µM Zn-Pheide, the uptake of the PS–HSA complex by the HUVEC cells might be difficult to identify, because at such conditions a significant fraction of gp60 will be occupied by free albumin. Therefore, we investigated the accumulation of Zn-Pheide in HUVECs at concentrations as low as 50 nM, and accordingly adjusted the concentrations of HSA, ranging from 10 nM to 12.5 µM. We observed that in the range of HSA concentrations from 0 to 1 μM, the accumulation of Zn-Pheide remained unchanged, while higher concentrations of the protein significantly reduced the PS level (Figure 4, grey bars). The photodynamic effect, in turn, increased with the increasing concentration of HSA, leading to a decrease in cell survival from 30% in the absence of HSA to 4% at 200 and 1000 nM of the protein, and then gradually decreased (Figure 4, black dots). This shows that the HSA–PS complex is internalized into the cells and its absorption causes an increase in the photodynamic activity of Zn-Pheide, probably due to the alteration of its cellular localization. Unfortunately, direct visualization of the PS in caveolae was infeasible, since it required permeabilization of the cells, which in turn caused the PS release.

**Figure 5:**
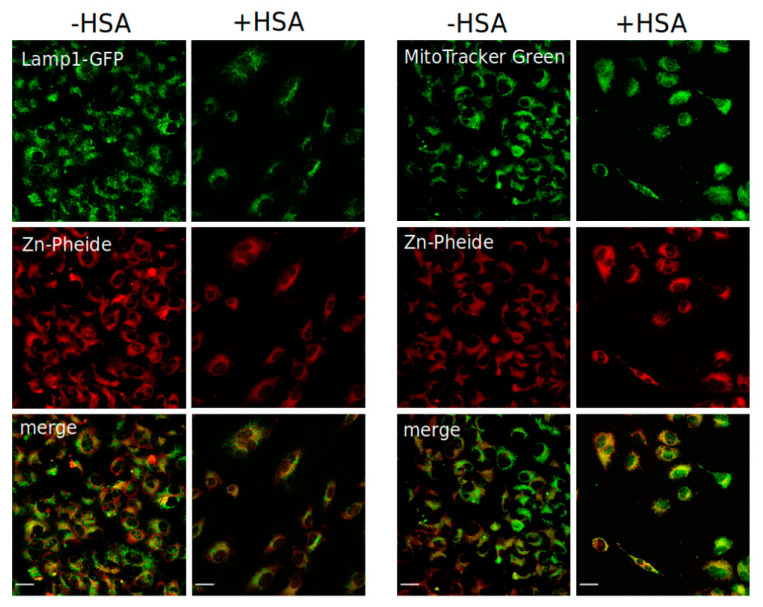
Colocalization analysis performed by confocal microscopy. The HUVEC cells stained with the CellLight™ Lysosomes-GFP, BacMam 2.0 reagent (fusion construct of Lamp1 and GFP) and Zn-Pheide (**left panel**), or with MitoTracker™ Green and Zn-Pheide (**right panel**), in the absence and presence of HSA. Maximum intensity projections of the representative 3D fluorescent images are shown. The scale bars represent 25 μm.

To evaluate the impact of HSA on the cellular localization of Zn-Pheide, we conducted a confocal microscopy study, the results of which are shown in Figure 5 and Figure S2 (Supplementary Materials). We observed a statistically significant increase in the lysosomal localization of Zn-Pheide in the presence of HSA compared to HSA-free conditions, while the mitochondrial localization of the PS remained unchanged. It should be noted that the increase in the lysosomal localization of Zn-Pheide could probably be greater at lower concentrations of the PS and HSA than used in the microscopic experiment, because in this case a smaller fraction of gp60 would be occupied by free HSA. However, such an experiment was not feasible to perform, since the detection of the PS by confocal microscopy required sufficiently high concentrations. Interestingly, we observed that in the absence of HSA, the fluorescence emitted by Zn-Pheide inside the cells appeared almost uniformly distributed in the covered area, while in the presence of HSA it was in the form of dots of varying intensity. This effect causes a visual impression that the PS colocalization with mitochondria is enhanced by HSA (Figure 5A, right panel), which, as shown by correlation analysis (Figure S2), was not the case.

**Figure 6:**
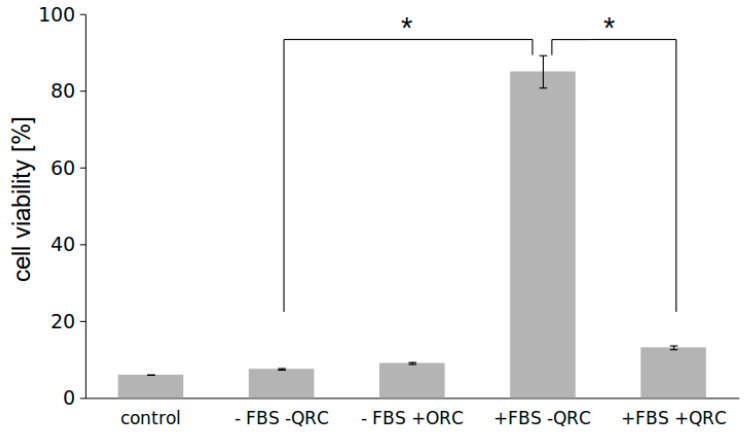
Viability (assessed by MTT assay) of HUVEC cells cultured on membrane inserts and allowed for the efflux of Zn-Pheide to either serum-depleted (-FBS) or serum-containing (+FBS) medium, in the presence or absence of quercetin (QRC). Cells that were not given time to remove the PS were indicated as control. Data are presented as mean ± SD from 3 inserts. Asterisks indicate significant differences between the groups, (*) indicates significant differences in cell viability between the groups, *p* < 0.05.

Once inside the endothelial cell, Zn-Pheide can leave it in two ways: either via BCRP, when it is in its free form, or by exocytosis, when it is either aggregated or complexed with albumin. Except for BCRP, probably no other transporter is involved in the efflux of free Zn-Pheide, as described in our previous paper [13]. At the same time, HUVEC cells cultured on plates are very imperfect models of the human endothelium, since they are unable to perform the transcytosis of albumin, which requires free space at both the luminal and abluminal sides of the cell [22]. Therefore, we conducted an experiment using HUVECs cultured on transwell inserts to enable the removal of the PS from both sides of the cells. After 3 h of incubation with the PS–HSA complex, the PS-containing medium was removed and replaced with fresh medium, either serum-free or supplemented with 2% fetal bovine serum. The cells were allowed for Zn-Pheide transport for the next 3 h and then illuminated, as described in the Materials and Methods section. The viability of cells that exported the PS to the serum-containing medium turned out to be significantly higher than that of the cells that were deprived of serum during the transport period (Figure 6). The presence of the BCRP inhibitor, quercetin, however, restored the cell susceptibility to PDT, decreasing their viability to the level only slightly higher compared to the FBS-free cells (Figure 6).

**Figure 7:**
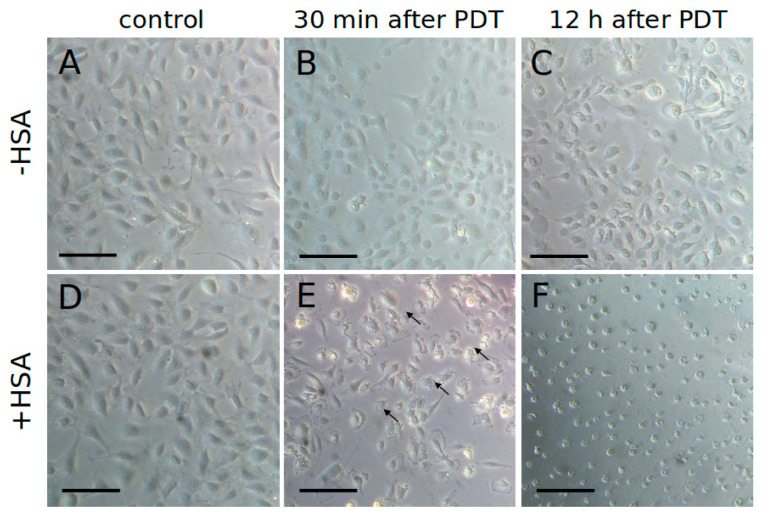
Morphology of HUVEC cells 30 min or 12 h after PDT. The cells accumulated Zn-Pheide (1 μM) in the absence (**A**–**C**) or presence (**D**–**F**) of HSA (250 μM). The control cells were incubated in the dark. The arrows indicate the apoptotic bodies formed after PDT performed in the presence of HSA. The scale bars represent 50 μm.

**Figure 8:**
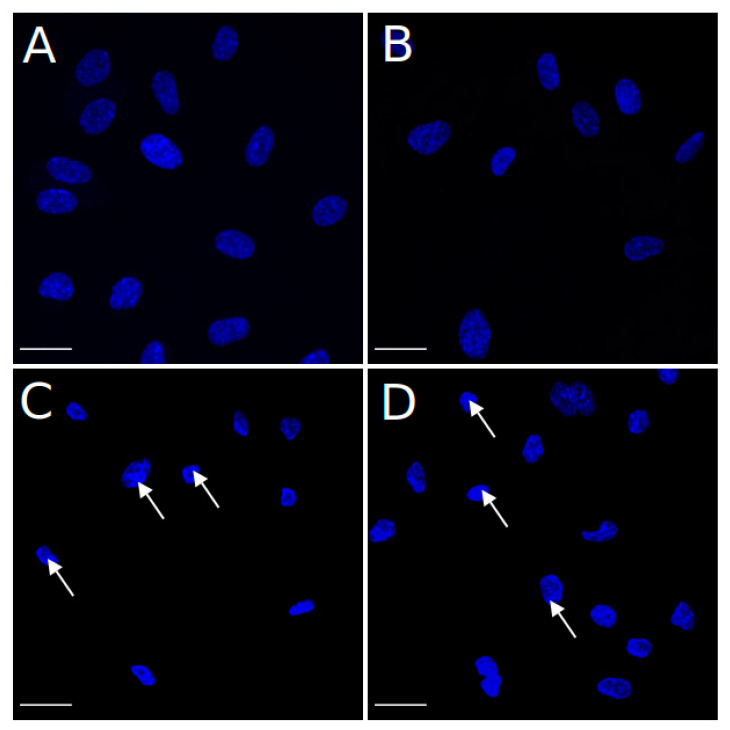
The HUVEC cells stained with Hoechst 33,342 to visualize the morphology of nuclei after PDT. (**A**) Control cells, (**B**) cells treated with 1 µM Zn-Pheide without HSA, (**C**) cells treated with 100 nM Zn-Pheide without HSA, and (**D**) cells treated with 1 µM in the presence of 250 µM HSA. The staining was performed 30 min after PDT. The arrows indicate the areas of condensed chromatin. The scale bars represent 25 μm.

We compared the morphology of HUVEC cells subjected to PDT after the incubation with 1 µM Zn-Pheide in the presence and absence of 250 µM HSA. In the cells treated with the Zn-Pheide–HSA complex, we observed a characteristic apoptotic morphology, including cell shrinkage, loss of adhesion, and the formation of apoptotic bodies (Figure 7E). When HSA was absent, in turn, the characteristic features of necrosis were observed, such as the increase in cell volume and plasma membrane blebbing (Figure 8B). These features were observed as early as 30 min after the PDT. After longer periods following PDT, we observed a significant shrinkage and detachment of the cells treated with the Zn-Pheide–HSA complex, while the cells treated solely with Zn-Pheide attached firmly to the culture vessel and appeared swollen, with plasma membrane blebs (Figure 7C). Importantly, no difference was observed between the control cells incubated with the PS in the dark with and without HSA (Figure 7A,D).

The observed differences in the morphology of PDT-subjected cells prompted us to confirm the type of cell death with additional methods: flow cytometric analysis with Annexin V/propidium iodide staining and confocal microscopy study after the staining of the nuclei with Hoechst 33342. In order to examine whether albumin has a direct effect on the type of cell death, or if its role is limited to the decrease of the PS absorption, in these experiments we included three groups of cells. The first group was incubated with 1 µM Zn-Pheide without HSA, the second with 100 nM Zn-Pheide also without HSA, and the third with 1 µM Zn-Pheide in the presence of 250 µM HSA. We chose the concentration of 100 nM, since it resulted in a similar intracellular level of Zn-Pheide, as in the case of the incubation performed with 1 µM Zn-Pheide in the presence of 250 µM HSA (data not shown). Additionally, the corresponding controls were prepared by the treatment of the cells with either HSA-free or HSA-containing culture medium with the appropriate concentration of DMSO. Since no visible differences were observed between these controls, we only present the results obtained for the cells incubated in the presence of HSA.

Staining with Hoechst revealed the shrinkage of nuclei (pyknosis) and chromatin condensation in the cells subjected to PDT after the incubation with 100 nM Zn-Pheide or 1 µM Zn-Pheide in the presence of HSA (Figure 8C,D). Counting the cells in which chromatin condensation was observed gave the same result of 66% apoptotic nuclei in both groups. Intranuclear chromatin condensation was widespread with a variable distribution. In some nuclei, clumps of chromatin were visible over their entire surface, while in others they were mostly adjacent to the nuclear membrane. These features were much less prominent in the cells treated with 1 µM Zn-Pheide (Figure 8B).

**Figure 9:**
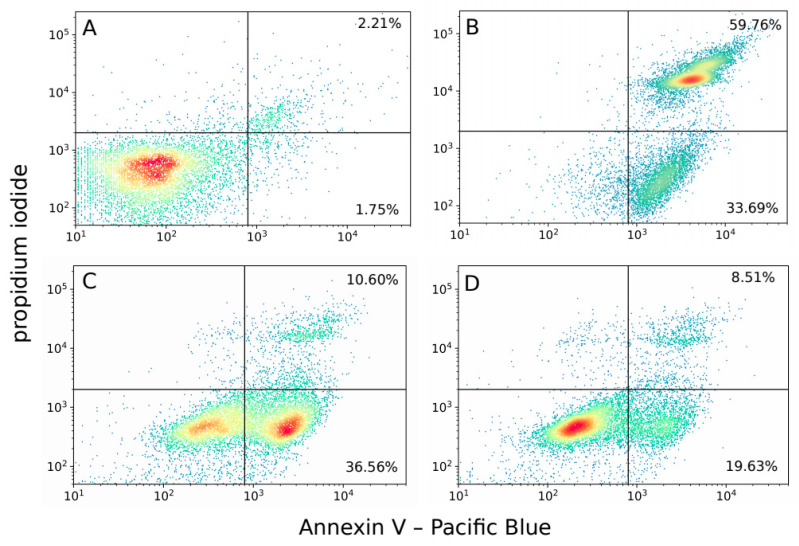
Flow cytometry analysis of HUVEC cells death mode after Zn-Pheide-induced PDT. The cells were stained with Pacific Blue-conjugated Annexin V and propidium iodide (PI), 30 min after irradiation. (**A**) Control cells, (**B**) cells treated with 1 µM Zn-Pheide without HSA, (**C**) cells treated with 100 nM Zn-Pheide without HSA, and (**D**) cells treated with 1 µM in the presence of 250 µM HSA. The percentage of cells undergoing early apoptosis (Annexin V—positive, PI—negative) and late apoptosis/necrosis (double-positive) is given.

The apoptotic/necrotic effect occurring as a consequence of the Zn-Pheide-induced PDT was further validated by cytometric analysis with Annexin V/propidium iodide double staining. Since, in the cytometric assay, similarly as in the nuclear staining assay, there was virtually no difference between the control samples prepared by incubating the cells in HSA-containing and HSA-free medium, we only present the result for the former control sample. In the group treated with 1 µM Zn-Pheide without serum, the majority of the cells were identified as late apoptotic/necrotic and very few living cells were present (Figure 9B). In contrast, among the cells treated with 100 nM Zn-Pheide or with the Zn-Pheide–HSA complex, a higher percentage of early apoptotic than late apoptotic/necrotic cells was observed (Figure 9C,D). Additionally, in the gate with low signal from both fluorophores, which is identified as living cells, the fluorescence of Annexin V is higher than in the control group. This suggests that these cells are likely to undergo apoptosis in a short period of time.

**Figure 10:**
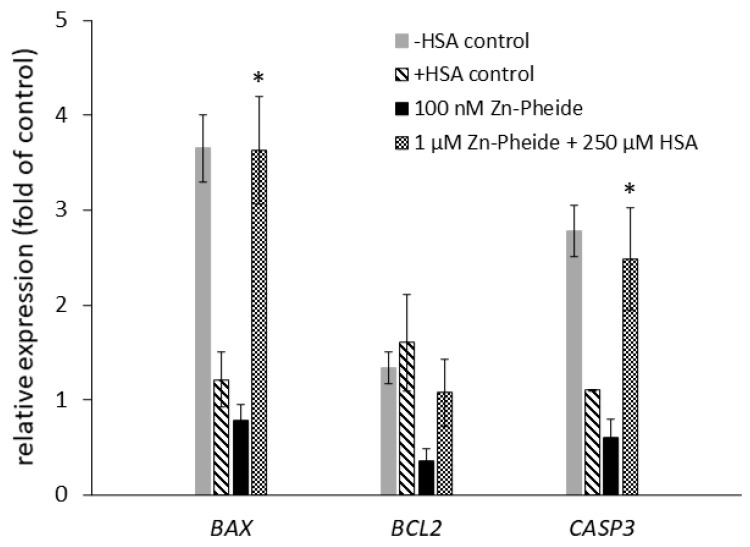
Relative quantification using real-time quantitative PCR of mRNA expression of *BAX*, *BCL2*, and *CASP3* genes in HUVEC cells upon Zn-Pheide-induced PDT. Data are shown as mean ± SD (*n* = 4). * *p* < 0.05 compared to HSA-containing control.

Interestingly, a higher percentage of early apoptotic cells was observed in the group treated with 100 nM Zn-Pheide than with the Zn-Pheide–HSA complex (Figure 9C,D), suggesting that the process of apoptosis occurs more rapidly in the former group. We suspected that this effect might result from differential pro- and antiapoptotic gene expression between these groups. Therefore, the expression of *BAX*, *BCL2*, and *CASP3* genes at the mRNA level was analyzed in HUVECs after Zn-Pheide-induced PDT. Gene expression was assessed 30 min after PDT using quantitative real-time PCR. In the group of cells treated with 1 µM Zn-Pheide in the presence of HSA, we observed an increase in the expression of *BAX* and *CASP3*, while the expression of *BCL2* remained unchanged (Figure 10). In contrast, the cells treated solely with 100 nM Zn-Pheide showed a decrease in expression of all three genes. Importantly, increased levels of *BAX* and *CASP3* mRNA were recorded in the control cells incubated in serum and the HSA-free medium, but not in the HSA-containing medium. This effect most likely results from the previously confirmed protective activity of albumin towards endothelial cells from apoptosis [23,24]. Importantly, in the cytometric study, we did not observe an increased number of apoptotic or necrotic cells in the HSA-free control compared to the HSA-containing control (data not shown). However, an increased expression of the *BAX* gene in the former may render these cells more susceptible to apoptosis, which consequently occurs more rapidly in the HSA-deprived cells than in the HSA-containing cells (Figure 9 C,D). This experiment shows that intracellular albumin has no effect on the PDT-induced expression of the analyzed genes, but pre-PDT serum starvation accelerates the induction of apoptosis.

## 3. Discussion

In the present study, we investigated the photodynamic effect induced with Zn-Pheide against HUVEC cells, including the type of cell death, particularly in the context of the binding of this PS to HSA. We found that Zn-Pheide induces a dose-dependent photodynamic effect in HUVECs with an IC50 value close to 20 nM, which is about 25-fold lower than the corresponding value for MCF-7 cells. At nanomolar concentrations, Zn-Pheide induces the death of HUVECs by apoptosis. The concentration of Zn-Pheide equal to 100 nM, which induces apoptotic cell death, at the same time kills almost 100% of the cells. Therefore, it is possible that the conditions of PDT can be adjusted to induce the death of the majority of endothelial cells by apoptosis. For comparison, low concentrations of Zn-Pheide (below 125 nM) had no effect on the viability of MCF-7 cells. In turn, increasing the concentration above this threshold leads to a rapid decrease in MCF-7 cell viability, which is observed as a steep dose–response curve. This difference in the susceptibility to Zn-Pheide-mediated PDT is interesting, particularly regarding that, in the case of a widely known photosensitizer, Photofrin, already approved for clinical use, equal sensitivity of endothelial and tumor cells to PDT was observed [25]. We hypothesize that the resistance of MCF-7 cells to Zn-Pheide-induced apoptosis may be, similarly as it is in the case of other treatment types, due to the lack of caspase-3 expression and a persistent PDT-induced cell cycle arrest in the phase G2 [26,27,28]. Caspase-3 is considered the most important effector caspase, responsible for the cleavage of several proteins and DNA fragmentation [29,30]. Although other caspases have been reported to function in lieu of the lack of caspase-3, the efficiency of this substitution varies for different treatment types [31,32]. It should be noted that the MCF-7 response to Zn-Pheide-mediated PDT is similar as those of other cancer cells [10]. This means that the high susceptibility to Zn-Pheide-mediated PDT in general distinguishes endothelial cells from cancer cells, and thus makes the first ones a good target for treatment. In light of the above, possibly the previously observed excellent therapeutic effects achieved with Zn-Pheide were due to the vascular shutdown effect rather than the direct killing of tumor cells [10,11].

Apart from the concentration of a PS, its cellular localization is also a factor determining the type of PDT-induced cell death mode. It has been shown that PSs accumulating in mitochondria induce a faster apoptotic effect than those localized in lysosomes or cell membranes [33,34]. Possibly, this can also be the reason for a fast apoptosis observed in the HUVECs. Previous studies on the cellular localization of Zn-Pheide showed that, in cancer cells, it does not colocalize with mitochondria, whereas in the HUVECs, its mitochondrial localization, though relatively low, was observed [10].

We found that HSA-bound Zn-Pheide enters HUVEC cells, which promotes its lysosomal localization, thereby increasing its photodynamic activity. The protein component of the complex is most likely degraded in lysosomes, whereas free Zn-Pheide can be transported outside the cell as long as free albumin is present in the environment. The HSA–Zn-Pheide complex can be internalized into the cells via the gp60 receptor (albondin), but possibly also other receptors present on endothelial cells, such as gp18 or gp30. The latter two receptors are believed to be scavenger receptors, which bind modified albumin molecules and direct it for degradation [35]. Since HSA undergoes slight conformational changes upon Zn-Pheide binding, it seems likely that these receptors are involved in its internalization, after which the protein component of the complex is degraded in lysosomes [14]. On the other hand, however, since the uptake of Zn-Pheide significantly decreases at high concentrations of HSA, the involvement of gp60 cannot be excluded. It is also important to note that, even at the saturating concentration of HSA, Zn-Pheide is likely to enter the cells in its free form, which occurs transiently within the equilibrium of its binding to HSA. It should be noted that the effect induced by Zn-Pheide in cancer patients will be probably strictly dependent on their serum albumin level. In hypoalbuminemia, which is a condition commonly encountered in patient with solid tumors, the cellular level of Zn-Pheide may be higher, and thus the photodynamic effect stronger, possibly leading to necrosis rather than apoptosis of endothelium [36,37].

In the transport-PDT study using HUVECs cultured on membrane inserts, a significant difference in viability was observed between the cells transporting Zn-Pheide to the serum-free and serum-containing medium. This allows to conclude on the way of the PS removal from the cells. There is no reason to assume that the transcytosis of albumin requires the presence of serum in the abluminal space. At the same time, the transport of Zn-Pheide was significantly accelerated by serum. This suggests that Zn-Pheide is effluxed from the cells predominantly in its free form, via BCRP, since this type of transport is accelerated in the presence of serum/albumin [13]. Thus, Zn-Pheide present inside HUVECs is probably predominantly in its free form. This can be an effect of HSA degradation in lysosomes, as well as the uptake of the PS in its free form, which occurs transiently within the equilibrium of its binding to albumin.

Zinc pheophorbide a is structurally similar to another PS, palladium bacteriopheophorbide (Pd-BPheide, WST09), known under its trade name TOOKAD^®^. Palladium bacteriopheophorbide is a derivative of bacteriochlorophyll a, isolated from the photosynthetic bacteria *Rhodovolum sulfidophilum* [38]. TOOKAD^®^, together with its water soluble derivative, padeliporfin (WST11, TOOKAD^®^ Soluble), are now approved in Europe and Israel for the VTP of low-risk prostate cancer [5]. TOOKAD^®^ has a strong light absorption (ε ~ 10^5^ M^−1^ cm^−1^) in the near infrared region (λ = 763 nm), lying in the so-called therapeutic window of human tissue. In comparison, Zn-Pheide shows a similar absorption in its Q_y_ band, which is, however, localized at shorter wavelengths (λ_max_ ~ 670 nm) [9]. This gives Pd-BPheide a slight advantage over Zn-Pheide, since the light of higher wavelengths penetrates deeper into tissues [39]. TOOKAD^®^ is cleared from the circulation within hours and its soluble derivative within minutes [40,41]. The rapid clearance of these PSs can be considered an advantage in terms of patient safety; however, their retention time might be too short to perform several irradiations upon a single PS injection, which is possible with Zn-Pheide (mean t_1/2_ = 44 h) [42].

The quantum yield of singlet oxygen generation (Φ_Δ_) by TOOKAD^®^ has been shown to reach 1 in organic solvents and 0.5 in TX-100 micelles. The aqueous environment, in turn, significantly reduced its singlet oxygen yield, instead promoting the generation of superoxide and hydroxyl radicals [43]. TOOKAD^®^ Soluble in water did not generate even traces of singlet oxygen [44]. In comparison, Φ_Δ_ of Zn-Pheide was equal to 0.49 in ethanol, 0.54 in F-127 micelles, 0.39 in DPPC, and 0.13 in water [45]. Superoxide anion generated by Zn-Pheide was also detected in water containing 5% of DMSO [46]. Similar values of Φ_Δ_ in micelles suggest that both PSs will be equally efficient in the generation of singlet oxygen in biological membranes. In the cytoplasm (water), in turn, TOOKAD^®^ will act almost solely via the type I reaction, and Zn-Pheide via both type I and type II.

Solubility in water, expressed by the octanol/water partition coefficient, is similar for TOOKAD^®^ (24) and Zn-Pheide (32) [38,41]. The former, however, is believed to require a nonionic surfactant, Cremophor, at the stage of administration, while the latter was administered to animals both with and without Cremophor, giving satisfactory treatment results in both cases [11]. This could result from a higher affinity of Zn-Pheide to serum proteins, and possibly also lipoproteins, which promotes its disaggregation and at the same time increases its retention time.

Although the exchange of the Mg ion for the Zn ion increases the stability of chlorophylls, Zn-Pheide shows a relatively high yield of photobleaching in water (0.55). This value is significantly decreased in ethanol (0.01) [45]. In comparison, the photobleaching of TOOKAD^®^ was also observed in methanol and acetone [47]. Complexation with albumin is likely to increase the photostability of both PSs, as was similarly observed for hematoporphyrin [48]. Moreover, since Zn-Pheide has a stronger bind to albumin than Pd-BPheide, the complexation of the former may increase its stability to a greater extent. This hypothesis, however, requires experimental confirmation.

Both Pd-BPheide and Zn-Pheide can be monitored in vivo for their proper dosimetry [11,49]. In the case of the former, it is, however, more difficult due to the very weak fluorescence and the low affinity to albumin, since the fluorescent signal from the albumin-bound PS is enhanced, as we showed for Zn-Pheide [14]. An additional factor that hinders the monitoring of TOOKAD^®^ is its very short retention time, which, in the case of Zn-Pheide, is much longer [42].

To the best of our knowledge, the interaction of WST09 and WST11 with BCRP has not been analyzed. Since these PSs are derived from a natural compound of a tetrapyrrole structure (bacteriochlorin), they are possibly ABCG2 substrates. However, as long as this hypothesis is not confirmed, the BCRP inhibitors cannot be used to prolong their retention time in tissues.

Experiments similar to those performed in the present study were conducted for WST11 with the usage of the murine endothelial cell line H5V. The LC50 value of WST11 determined in the absence of serum at the 2 h incubation time and the light dose of 12 J/cm^2^ was as high as 1 μM [41]. In comparison, the LC50 value of Zn-Pheide against HUVECs was only 20 nM at 3 h accumulation time and the light dose of 2 J/cm^2^.

It has been shown that WST11, similarly to Zn-Pheide, is partially incorporated into the cells via receptor-dependent endocytosis. However, given that the association constant of WST11 to albumin (K_a_ ~ 10^4^ M^−1^) is two orders of magnitude lower than the corresponding value for Zn-Pheide, the role of albumin in trafficking the PS into the endothelium is probably more significant for the latter. Similarly, as in the case of Zn-Pheide, both the serum and the pure albumin accelerated the removal of WST11 from the cells. This indicates that WST11 leaves the cells in its free form, possibly via BCRP [13,41].

A drawback of bacteriochlorophyllide-based PSs is their high production cost [50]. This does not apply to Zn-Pheide, which is produced from chlorophyll a, available in virtually unlimited quantities, and which does not require complex chemical modifications [10].

In summary, since the applicability of a PS for therapy is determined by a number of features, it is difficult to state unequivocally whether Zn-Pheide is superior to TOOKAD^®^ in terms of its application in VTP. Undoubtedly, however, it possesses several characteristics that make it a noteworthy candidate for a potential use in both the vascular-targeted treatment of solid tumors, as well as a range of vascular abnormalities.

## 4. Materials and Methods

### 4.1. Photosensitizer

Zinc pheophorbide a (Zn-Pheide) was obtained from pheophorbide a (Cayman Chemical, Ann Arbor, MI, USA) via direct metalation with zinc acetate in methanol at 50 °C and purified, as described earlier [10]. Purity of the compound according to HPLC measurement was at least 96%. The concentration of Zn-Pheide was determined spectrophotometrically in ethanol, using the extinction coefficients at the Q_y_ band equal to 71,500 M^−1^ cm^−1^. Aliquots of the PS were stored dry at −20 °C under nitrogen atmosphere. For each experiment, fresh solutions of the PS were prepared by dissolving them in appropriate volumes of DMSO. All experiments were performed under dim light to avoid degradation of the PS and uncontrolled photodamage of the cells.

Complex of the PS with HSA was prepared by addition of the PS stock solution in DMSO to solution of albumin in PBS, pH 7.4, and incubation at room temperature for 10 min to reach the binding equilibrium.

### 4.2. Cell Lines

Human umbilical vein endothelial cells (HUVECs) pooled from multiple donors were obtained from PromoCell (Heidelberg, Germany). The cells were cultured in the endothelial cell growth medium (ECM, PromoCell, Heidelberg, Germany) supplemented with 2% fetal bovine serum (FBS), 4 µL/mL endothelial cell growth supplement, 0.1 ng/mL epidermal growth factor, 1 ng/mL basic fibroblast growth factor, 90 μg/mL heparin, and 1 μg/mL hydrocortisone. The human breast carcinoma cell line MCF-7 was obtained from the American Type Culture Collection (ATCC, Manassas, VA, USA). The cells were maintained in Dulbecco’s modified Eagle’s medium (DMEM, Thermo Fisher Scientific, Waltham, MA, USA) supplemented with 10% (*v*/*v*) FBS, 2 mM glutamine, 1 mM sodium pyruvate, penicillin (100 units/mL), and streptomycin (100 µg/mL). Both cell lines were cultured at 37 °C in a humidified atmosphere of 5% CO_2_.

### 4.3. Photodynamic Treatment and Cell Viability Assays

Cytotoxicity of Zn-Pheide, both in the dark and after irradiation, was determined by MTT assay. For cytotoxicity studies, 3 × 10^4^ cells were seeded onto 96-well plates. After an overnight growth, the cells were incubated in the dark for 3 h with Zn-Pheide at various concentrations. Control cells were incubated with the appropriate concentration of DMSO (below 0.5%) without the PS. For PDT, the cells were rinsed with PBS, covered with HBSS, and illuminated with 2 J/cm^2^ delivered by a LED illuminator equipped with a 600 nm cut-off filter. After that, they were covered with complete culture medium and incubated for another 24 h. Then, 0.5 mg/mL MTT (3-(4,5-dimethylthiazol-2-yl)-2,5-diphenyltetrazolium bromide), (Carl Roth, Karlsruhe, Germany) was added to each well, and the cells were incubated for another 3 h. The precipitated formazan crystals were dissolved in an ethanol/DMSO mixture (*v*/*v*, 1:1) and the absorbance of the solutions at 570 nm was measured using a SpectraMax i3 plate reader (Molecular Devices, San Jose, CA, USA). The signal from the treated cells was compared to that of the vehicle-only control cells (100%) to calculate the percentage viability. All experiments were performed in triplicates. Dose–response curves were fitted and IC50 values determined in Origin 2021 [51].

### 4.4. Cellular Localization of Zn-Pheide

Cellular localization of Zn-Pheide in HUVEC cells in the presence or absence of HSA was assessed by confocal microscopy using single organelle staining. Lysosomes were labelled using CellLight™ Lysosomes-GFP, BacMam 2.0 reagent (Thermo Fisher Scientific, Waltham, MA, USA) according to the manufacturer’s protocol. Then, the cells were incubated with either 0.5 µM Zn-Pheide alone for 2 h or with 1 µM Zn-Pheide and 250 µM HSA overnight. After removal of the PS, mitochondria were stained with 100 nM MitoTracker™ Green (Thermo Fisher Scientific, Waltham, MA, USA) for 45 min. Then, the cells were washed with PBS and fixed with 4% paraformaldehyde in PBS at room temperature for 10 min. Confocal images were collected using an upright confocal microscope Leica SP8. Stacks of confocal 12-bit images with a voxel size of 0.186 × 0.186 × 0.999 μm were acquired using oil 40× objective (HC PL APO CS2 40×/1.3 OIL). The organelle markers were excited with laser 488 nm, while the Zn-Pheide was excited with laser 647 nm. The emission was collected in the range 495–590 nm for the organelle markers, and 643–730 nm for Zn-Pheide. The acquisition was performed in sequential mode. Colocalization analysis was performed using the JACoP plugin tool in Fiji ImageJ to calculate the Pearson’s correlation coefficients [52,53].

### 4.5. Efflux Assay

The HUVECs were seeded onto PET ThinCert^®^ cell culture inserts with 0.4 µm pores (Greiner Bio-One, Kremsmünster, Austria, cat. no. 665640) at a density of 10^5^ of cells per insert and left for attachment. Next day, Zn-Pheide at a concentration of 1 µM in the presence of 250 µM HSA was applied to both top and bottom chambers. After 3 h of incubation, the PS-containing medium was replaced with either serum-free or complete ECM medium (containing 2% FBS) in both chambers. The cells were incubated for the next 3 h for the PS efflux and then irradiated with 2 J/cm^2^ of red light. Control (100% viability) cells were prepared by incubation of the cells without Zn-Pheide and with or without serum, respectively. Additional control, for assessment of the PS efflux rate, was prepared by illuminating the cells right after the incubation with PS-HSA complex, without the transport period. The experiments were performed in triplicates and in the presence or absence of BCRP inhibitor, quercetin, at a concentration of 100 µM. The viability of the cells was examined by MTT assay 24 h after the PDT.

### 4.6. Hoechst Staining Assay

The HUVEC cells were seeded onto 10 mm glass slides (approximately 2.5 × 10^4^ cells per well) and cultured overnight. Photodynamic treatment was performed after 3 h of cell treatment either with sole 1 µM and 100 nM Zn-Pheide in serum-free culture medium or 1 µM Zn-Pheide in the presence of 250 µM HSA. Control cells were incubated in serum-free ECM medium, with or without 250 µM HSA, containing an appropriate concentration of DMSO. Thirty minutes after the PDT, the cells were fixed with 4% paraformaldehyde for 10 min at room temperature, washed twice with PBS, and stained with 5 µg/mL Hoechst 33,342 for 10 min at room temperature. Then, they were washed again, mounted onto microscope slides, and imaged using an inverted confocal microscope Zeiss Cell Observer. Stacks of confocal 16-bit images with a voxel size of 0.167 × 0.167 × 0.24 μm were acquired using oil 40× objective (Plan Apochromat 40×/1.4 OIL DIC (UV) Vis-IR M27), exposure time 100 ms, and depth of focus 0.72 μm. Hoechst 33,342 was excited with laser 405 nm. The emission was collected using filters: RQFT 405/488/568/647 and BP 450/50 nm. ImageJ (Fiji) was used for image analysis [52].

### 4.7. Analysis of Cell Death Mode by Flow Cytometry

The HUVEC cells were seeded onto a 6-well plate (2.5 × 10^5^ cells per well) and cultured overnight. Next day, PDT was performed after 3 h of cell treatment with either sole 1 µM and 100 nM Zn-Pheide in serum-free culture medium or 1 µM Zn-Pheide in the presence of 250 µM HSA. Control cells were incubated in serum-free ECM medium, with or without 250 HSA, containing an appropriate concentration of DMSO. Heat-treated cells (55 °C for 20 min) served as a positive control. Thirty minutes after the PDT, the cells were harvested by trypsinization, washed with PBS, and suspended in Annexin V-binding buffer (10 mM HEPES pH 7.4, 140 mM NaCl, 2.5 mM CaCl_2_) at a density of 10^6^ cells/mL. The cells were stained with Annexin V, Pacific Blue™ conjugate (Thermo Fisher Scientific) and propidium iodide (BD Biosciences, Franklin Lakes, NJ, USA) for 15 min at room temperature. Then, after 5-fold dilution with Annexin V-binding buffer, the cells were analyzed using a BD FACSCanto II flow cytometer. Data were processed using the BD FACSDiva 8.0.1 and FlowCal software [54].

### 4.8. Quantitative Real-Time PCR

For the analysis of pro- and antiapoptotic genes expression in PDT-subjected cells, HUVECs were seeded on a 6-well plate (3 × 10^5^ cells per well) and cultured overnight. Next day, the cells were treated with either 100 nM Zn-Pheide in serum-free medium or with 1 µM Zn-Pheide in the presence of 250 µM HSA, for 3 h. Control cells were incubated in serum-free ECM medium, with or without 250 µM HSA, containing an appropriate concentration of DMSO (below 0.5%). Then, the cells were irradiated as described earlier. Thirty minutes after the PDT, total RNA was isolated from the cells using the GeneMATRIX Universal RNA kit (EURx, Gdańsk, Poland). The RNA concentration was measured using a NanoDrop 8000 spectrophotometer (Thermo Fisher Scientific). Reverse transcription was performed using the NG dART RT kit (EURx, Gdańsk, Poland) with oligo(dT) primer. Quantitative real time PCR was performed using the iProof™ High-Fidelity DNA Polymerase (Bio-Rad, Hercules, CA, USA), together with SYBR Green dye (Sigma-Aldrich, St. Louis, MO, USA) and the primers listed in Table 1. The analysis was performed using a Rotor Gene Q thermocycler (Qiagen, Hilden, Germany). The relative fold change in the mRNA expression of the target gene was quantified using the 2^−ΔΔCt^ method. Glyceraldehyde 3-phosphate dehydrogenase (GAPDH) was used as a housekeeping gene. Nontreated cells were used as a calibrator for quantification.

For the determination of ABCG2 expression at the mRNA level, total RNA was isolated from untreated MCF-7 and HUVEC cells and reverse transcription was performed as described above. Quantitative real-time PCR was performed using human ABCG2-specific TaqMan^®^ system (Thermo Fisher Scientific). The expression was determined by the absolute quantification using a standard curve generated on the basis of an ABCG2 gene containing plasmid construct.

### 4.9. Statistical Analysis

To assess statistical significance of differences between two sets of data, the two-sided unpaired Student’s *t*-test was conducted in Origin 2021. The *p*-values < 0.05 were considered statistically significant as indicated by asterisks.

## 5. Conclusions

In the present study, the in vitro effects of Zn-Pheide-based PDT on human umbilical vein endothelial cells (HUVECs) were determined through viability and cell death mode assays. We compared HUVECs with MCF-7 cells in terms of their susceptibility to Zn-Pheide-induced PDT, showing that the 50% inhibitory concentration (IC50) is roughly 25 times lower for the former line. We have also shown that nanomolar concentrations of Zn-Pheide combined with low doses of light effectively kill HUVECs by apoptosis, whereas concentrations as low as 1 µM induce their death primarily by necrosis. Additionally, we found that albumin-complexed Zn-Pheide enters HUVECs and increases the efficacy of PDT, probably by directing the PS to lysosomes. Based on photodynamic experiments performed with the use of culture inserts, we also showed that albumin complexed with Zn-Pheide is apparently degraded in HUVECs, whereas free Zn-Pheide can leave the cells via BCRP-mediated transport.

Additionally, we compared Zn-Pheide with a clinically approved photosensitizer, TOOKAD^®^, in terms of their photophysical and pharmacokinetic properties, showing that the former may serve as a valuable alternative to the latter.

The results obtained contribute to a better understanding of the mechanism of action of Zn-Pheide and demonstrate that it is a promising PS for the VTP of solid tumors and possibly a range of vascular anomalies.

## Figures and Tables

**Table 1:** Primers used for quantitative real-time PCR.

Gene	Forward Primer Sequence (5′→3′)	Reverse Primer Sequence (5′→3′)
*BAX*	AGTGGCAGCTGACATGTTTT	GGAGGAAGTCCAATGTCCAG
*BCL2*	GCCCTGTGGATGACTGAGTA	GGCCGTACAGTTCCACAAAG
*CASP3*	TGTGAGGCGGTTGTGGAAGAGT	AATGGGGGAAGAGGCAGGTGCA
*GAPDH*	CGGAGTCAACGGATTTGGTCGTAT	AGCCTTCTCCATGGTGGTGAAGAC

## Data Availability

The data are contained within the article and Supplementary Materials.

## Supplementary Materials

The following supporting information can be downloaded at: https://www.mdpi.com/article/10.3390/ph15020235/s1, Figure S1. Dark cytotoxicity of Zn-Pheide against MCF-7 and HUVEC cells in the absence of albumin; Figure S2. Correlation analysis of Zn-Pheide colocalization in lysosomes and mitochondria in the absence and presence of HSA; Figure S3. Absorption and emission spectra of Zn-Pheide recorded in ethanol; Figure S4. Positive controls included in the experiments.
