# Supplementary material for: Zinc-Substituted Pheophorbide A Is a Safe and Efficient Antivascular Photodynamic Agent
Source: Pharmaceuticals (Basel). 2022 Feb 16;15(2):235. doi: 10.3390/ph15020235 (PMC8874758; doi:10.3390/ph15020235)
Supplement: Supplementary file 1 [file pharmaceuticals-15-00235-s001.zip › pharmaceuticals-1576068-supplementary.pdf]

Supplementary materials

Zinc-substituted pheophorbide a is a safe and efficient antivascular photodynamic agent

Milena J. Szafraniec, Monika Toporkiewicz, Andrzej Gamian

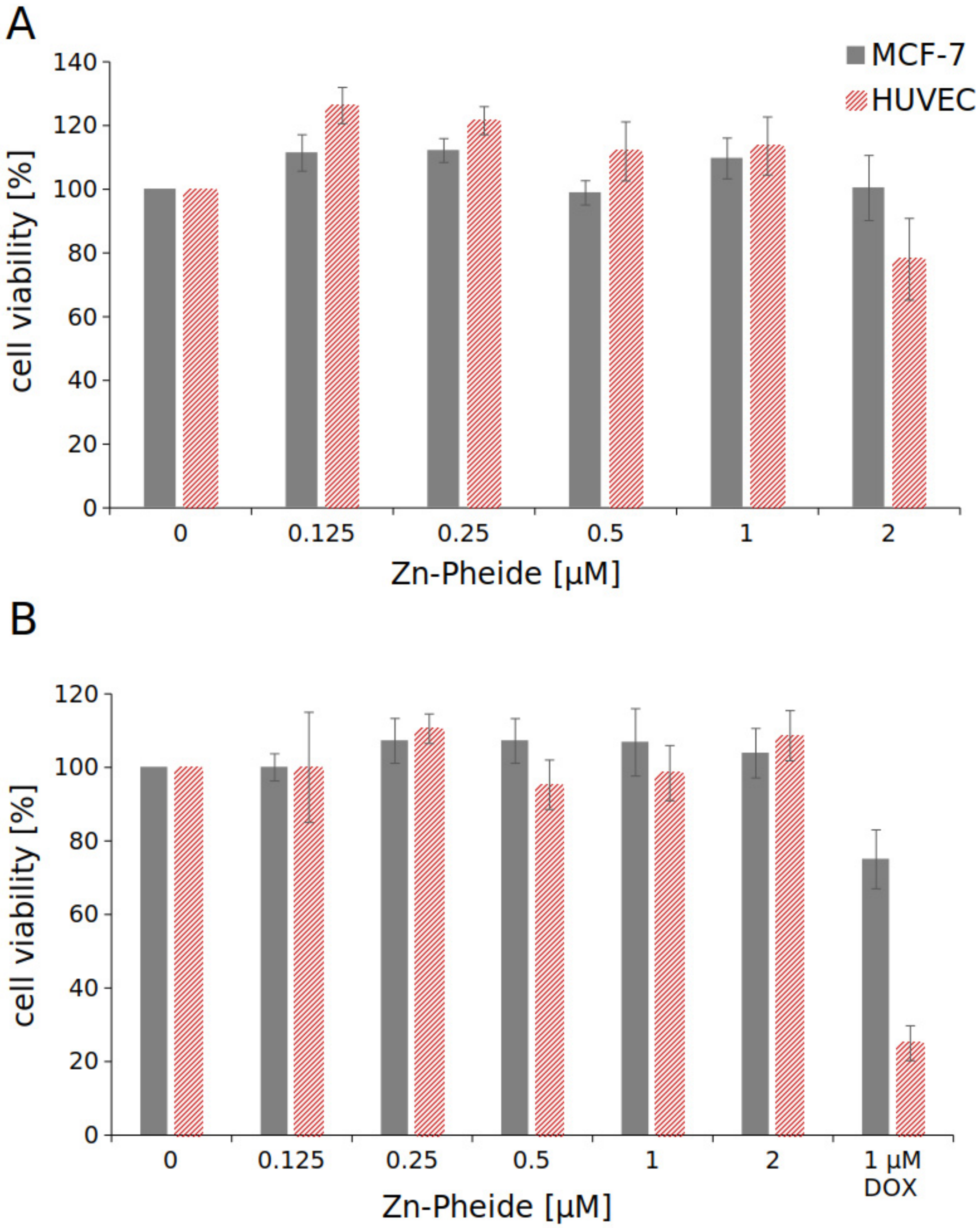

Figure S1. Dark cytotoxicity of Zn-Pheide against MCF-7 and HUVEC cells in the absence of albumin (**A**) and in the presence of 250 µM HSA (**B**). Cells treated with appropriate concentration of DMSO without the PS served as negative controls and were assigned 100% viability. Cells treated with doxorubicin (DOX) at a concentration of 1 µM served as positive controls. Analysis performed by MTT assay after 3 h of incubation. The results are shown as mean ± SD, n= 3.

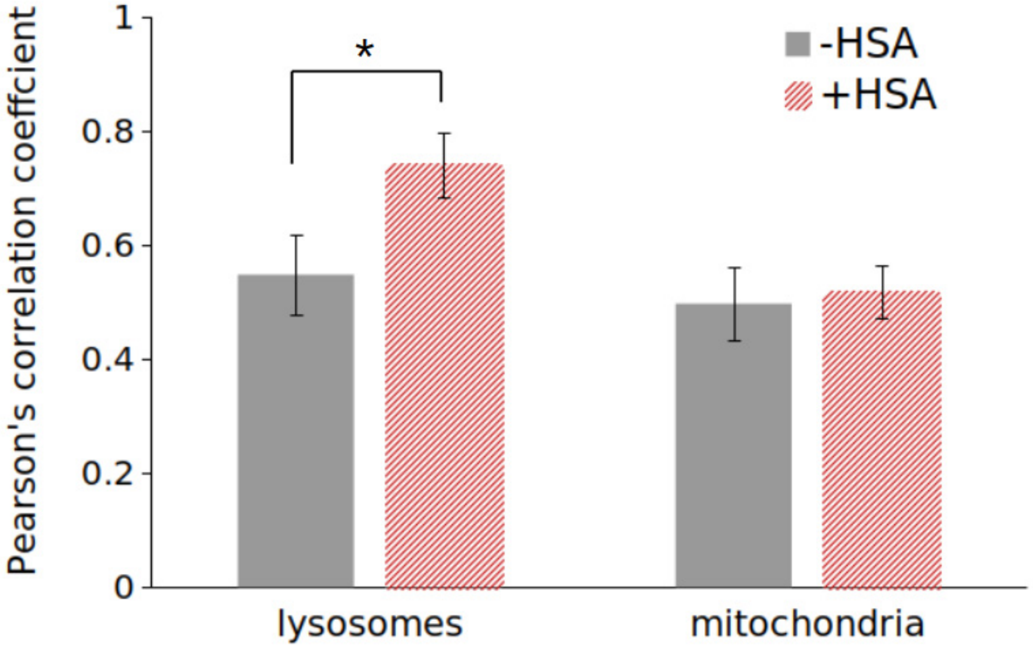

Figure S2. Correlation analysis of Zn-Pheide colocalization in lysosomes and mitochondria in the absence and presence of HSA. The graph shows the Pearson's correlation coefficients determined with the JACoP plugin from the ImageJ software. Significant differences between HSA-free and HSA-containing samples were indicated by asterisk (*), $p < 0.05$.

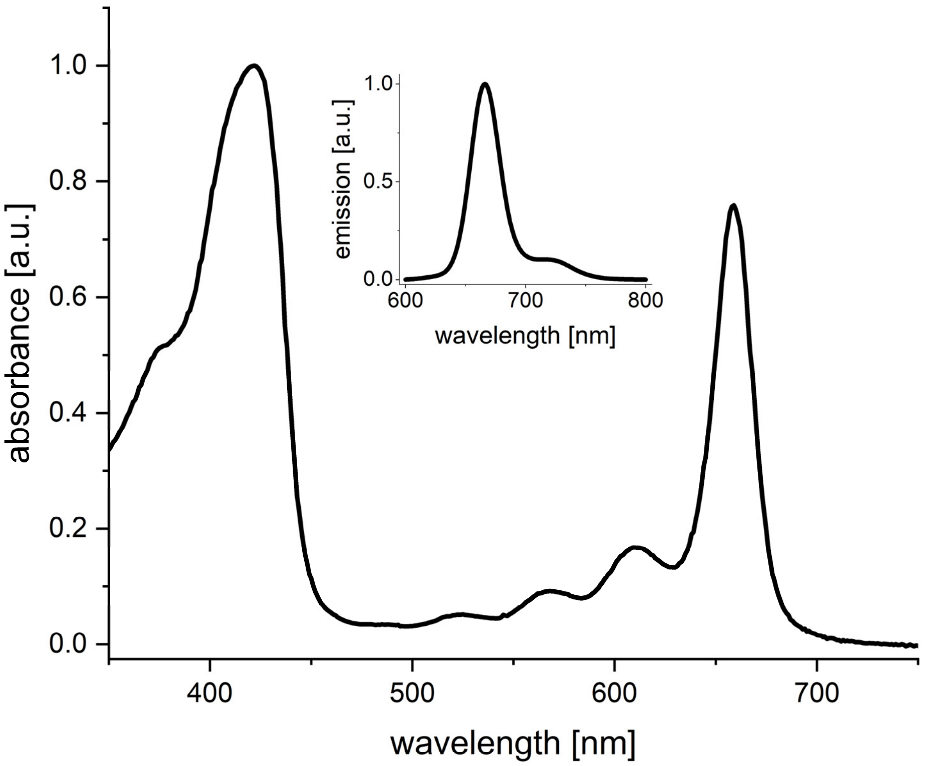

Figure S3. Absorption and emission (inset) spectra of Zn-Pheide recorded in ethanol. The emission spectrum was measured after excitation in the Soret band (430 nm).

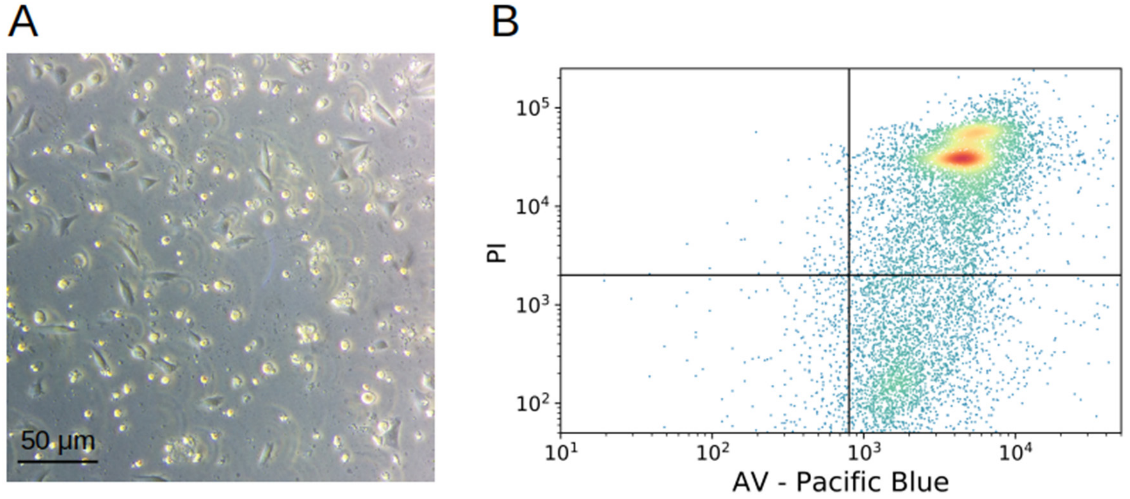

Figure S4. Positive controls included in the experiments: (**A**) HUVEC cells treated with 1 µM doxorubicin for 24 h, (**B**) Annexin V/PI staining of heat-treated HUVEC cells (incubation at 55 °C for 20 min).
